# Prevalence of Hypertension, Treatment, and Blood Pressure Targets in Canada Associated With the 2017 American College of Cardiology and American Heart Association Blood Pressure Guidelines

**DOI:** 10.1001/jamanetworkopen.2019.0406

**Published:** 2019-03-08

**Authors:** Stephanie Garies, Sylvia Hao, Kerry McBrien, Tyler Williamson, Mingkai Peng, Nadia A. Khan, Raj S. Padwal, Hude Quan, Alexander A. Leung, for Hypertension Canada’s Research and Evaluation Committee

**Affiliations:** 1 Department of Community Health Sciences, University of Calgary, Calgary, Alberta, Canada; 2 Department of Family Medicine, University of Calgary, Calgary, Alberta, Canada; 3 Libin Cardiovascular Institute of Alberta, University of Calgary, Calgary, Alberta, Canada; 4 Division of General Internal Medicine, University of British Columbia, Vancouver, British Columbia, Canada; 5 Centre for Health Evaluation and Outcome Sciences, Vancouver, British Columbia, Canada; 6 Department of Medicine, University of Alberta, Edmonton, Alberta, Canada; 7 Department of Medicine, University of Calgary, Calgary, Alberta, Canada

## Abstract

**Importance:**

The 2017 American College of Cardiology and American Heart Association (ACC/AHA) blood pressure (BP) guidelines redefined hypertension using a BP threshold of 130/80 mm Hg or greater and applied a treatment target of less than 130/80 mm Hg.

**Objective:**

To evaluate the potential change in the diagnosis, treatment, and control of hypertension in a Canadian cohort of patients with hypertension attending primary care practices using the ACC/AHA guidelines.

**Design, Setting, and Participants:**

This cross-sectional study used primary care practices across Canada electronic medical record data from the Canadian Primary Care Sentinel Surveillance Network, extracted as of June 30, 2015. Adults with at least 1 primary care encounter in the previous 2 years (July 1, 2013, to June 30, 2015) were included in the study. Those with current hypertension were identified using a validated definition consisting of diagnoses, billing codes, and/or antihypertensive medication from within the primary care electronic medical record. Data analysis was conducted from December 2017 to July 2018.

**Main Outcomes and Measures:**

Proportion of individuals with a diagnosis of hypertension, prescribed antihypertensive medication, and meeting treatment BP targets.

**Results:**

Of the 594 492 Canadian participants included in the study, 144 348 (24.2%) had hypertension (45.6% male; mean [SD] age, 65.5 [14.5] years). On applying the ACC/AHA guidelines, 252 279 individuals (42.4%) were considered hypertensive and half (51.0%; 95% CI, 50.8%-51.2%) were prescribed an antihypertensive medication. Individuals who were not previously considered to have hypertension but were reclassified as having elevated BP using the lower cutoff of 130/80 mm Hg or greater tended to be younger and were at lower cardiovascular risk. There was a shift toward more individuals requiring antihypertensive treatment, particularly in the lower-risk categories. The crude prevalence of hypertension increased from 13.3% to 32.0% in those aged 18 to 64 years, and of those aged 65 years and older, 16.6% more individuals were reclassified as having hypertension (from 55.2% to 71.8%). Only 12.3% of those who were considered at high risk were reclassified as hypertensive.

**Conclusions and Relevance:**

Adoption of the ACC/AHA BP guidelines would result in a near doubling in the prevalence of hypertension in Canada. The changes would largely affect individuals who are younger and at low to moderate cardiovascular risk.

## Key Points

**Question:**

What would be the projected change in the diagnosis and treatment of hypertension in Canada using the American College of Cardiology and American Heart Association blood pressure guidelines?

**Findings:**

This cross-sectional study of 594 492 individuals in Canada estimated that lowering the diagnostic blood pressure cutoff to 130/80 mm Hg or greater and targeting less than 130/80 mm Hg would nearly double the crude prevalence of hypertension in Canadian adults (from 24.2% to 42.4%), mostly affecting individuals at low to moderate cardiovascular risk.

**Meaning:**

Redefining hypertension may result in a considerable change in the epidemiology of hypertension in Canada.

## Teaser

This cross-sectional study evaluates the potential change in the diagnosis, treatment, and control of hypertension in a Canadian cohort of patients with hypertension attending primary care practices using the 2017 American College of Cardiology and American Heart Association (ACC/AHA) guidelines.

## Introduction

The 2017 American College of Cardiology and American Heart Association (ACC/AHA) blood pressure (BP) guidelines introduced a number of striking changes to the diagnosis and management of hypertension.^1^ These guidelines redefined hypertension according to BP levels 130/80 mm Hg or greater and revised the BP target to less than 130/80 mm Hg.^1^ These recommendations were largely based on observational data reporting a linear association between BP and coronary heart disease, stroke, and death, even with BP levels as low as 120-129/80-89 mm Hg,^2,3,4,5,6^ as well as the Systolic Blood Pressure Intervention Trial (SPRINT) which reported a reduction in major cardiovascular events with an intensive systolic BP target less than 120 mm Hg compared with less than 140 mm Hg.^7^ The response to the ACC/AHA guideline recommendations has been controversial.^8,9^ Accordingly, many international panels have maintained a BP diagnostic threshold of 140/90 mm Hg or greater and a target of less than 140/90 mm Hg for individuals at low to moderate cardiovascular risk.^10,11,12^

The proposed changes from the ACC/AHA can potentially be associated with changes to the epidemiology of hypertension, in addition to resource use, policy, and health care delivery.^13,14,15,16,17,18,19^ The adoption of the ACC/AHA guidelines in the United States is projected to raise the prevalence of hypertension to 45.6% of adults.^20^ In addition, pharmacological treatment would be recommended for 4.2 million treatment-naive individuals and another 7.9 million people may require treatment intensification.^20^

Canada has a slightly lower prevalence of hypertension at approximately 23%^21^ compared with 30% in the United States and England^22,23^ and leads the world in rates of national hypertension control.^21,22^ Even so, a downward shift in BP diagnostic thresholds and treatment targets may produce a surge in hypertension cases, creating challenges in an already overburdened publicly funded health care system with limited resources, such as Canada. This study aimed to quantify the proportion of adult Canadians potentially affected by the ACC/AHA BP guidelines.

## Methods

### Data Source

The Canadian Primary Care Sentinel Surveillance Network (CPCSSN) maintains a national primary care electronic medical record (EMR) database, with approximately 1400 family physicians and nurse practitioners contributing data for approximately 1.8 million primary care patients across the country.^24^ The EMR data are extracted, cleaned, and processed biannually, then standardized and merged into a national repository.^24^ These data include patient demographics, diagnoses (both text and *International Classification of Diseases, Ninth Revision *codes), prescribed medications, laboratory results, physical measurements (eg, blood pressure, height, and weight), behavioral risk factors, physician billing claims, and medical procedures. The national CPCSSN data have been found to be slightly overrepresented by adults 65 years and older as well as females compared with the general population, although this is reflective of a typical population attending primary care.^25^ For this cross-sectional study, national CPCSSN data extracted up to June 30, 2015, were used and the analysis was conducted from December 2017 to July 2018. The base sample included all adult patients (age ≥18 years) who had at least 1 primary care visit in the previous 2 years (July 1, 2013, to June 30, 2015), which is commonly used to define the active practice population.^25^ The earliest date of data varies for each patient, depending on when they first attended the clinic or when the clinic implemented an EMR system; this base sample contained a median of 6 years of longitudinal clinical data per patient (interquartile range, 5 years). A waiver of individual patient consent was granted by the research ethics boards at each participating site. For this study, ethics approval was obtained from the Conjoint Health Research Ethics Board at the University of Calgary. This study was reported according to the Strengthening the Reporting of Observational Studies in Epidemiology (STROBE) reporting guideline.

### Hypertension Diagnosis, Treatment Initiation Thresholds, and BP Targets

A case-finding algorithm specific to primary care EMR data was used to identify individuals currently diagnosed with hypertension, using relevant *International Classification of Diseases, Ninth Revision* codes and text words in the patient medical record as well as prescriptions for antihypertensive drugs.^26^ This algorithm has been validated with a sensitivity of 84.9% and a specificity of 93.5% for identifying people with hypertension in the CPCSSN database.^26^ To estimate the number of people who would potentially be reclassified as having hypertension according to the 2017 ACC/AHA guidelines, we additionally considered individuals with a systolic BP (SBP) level 130 mm Hg or greater and/or diastolic BP (DBP) level 80 mm Hg or greater to have elevated BP, taken as a mean of 3 most recent BP readings in the previous 2 years. A treatment target of SBP levels less than 130 mm Hg and DBP levels less than 80 mm Hg was applied to everyone under the ACC/AHA guidelines. For the Canadian guidelines, a general target of SBP levels less than 140 mm Hg and DBP levels less than 90 mm Hg was used, except for those with diabetes (SBP <130 mm Hg and DBP <80 mm Hg).^10^

### Measures and Disease Definitions

Blood pressure measurements were extracted from the EMRs of community primary care professionals and likely reflected a variety of BP measurement techniques (eg, attended vs unattended) and devices (eg, automated vs manual). To estimate the number of people with hypertension according to the ACC/AHA cutoff, an average of the 3 most recent BP recordings in the previous 2 years was used. If less than 3 BP values were present, either an average of 2 BP measurements or a single reading was used, depending on what was available, as in previous studies.^27^ The most recent BP value was used to assess BP targets.

Most recent laboratory values were used to assess the presence of certain comorbid conditions: chronic kidney disease (estimated glomerular filtration rate <60 mL/min/1.73 m^2^); dyslipidemia (low-density lipoprotein cholesterol ≥193 mg/dL [to convert to millimoles per liter, multiply by 0.0259]); and albuminuria (urine albumin-to-creatinine ratio >265 mg/g [>30 mg/mmol]). Diabetes was identified using a validated CPCSSN case definition, which included a combination of text words, diagnostic codes, fasting glucose, hemoglobin A_1c_, and diabetes-related medication.^26^ The presence of other related comorbidities (eg, cardiovascular disease [CVD], peripheral arterial disease, stroke, congestive heart failure, retinopathy, and vascular dementia) was determined by searching the relevant text words and *International Classification of Diseases, Ninth Revision* diagnosis codes in the EMR data using previously published definitions or clinician expertise.^28,29,30,31^ Current smoking status was ascertained from the risk factor data available in the EMR.

Medications in the CPCSSN database correspond with prescriptions issued by the patient’s primary care professional. The CPCSSN assigns codes from the Anatomical Therapeutic Chemical classification system to each medication.^32^ The antihypertensive drugs used in the CPCSSN definition were augmented to include a more current list of medications, which were defined with the following Anatomical Therapeutic Chemical codes: β-blockers, C07 (excluding C07AA04, C07AA07, C07AA12, and C07AG02); renin-angiotensin system inhibitors, C09; diuretics, C03 (excluding C03BA08 and C03CA01); calcium channel blockers, C08; and other antihypertensive drugs, C02 (excluding C02KX01), C10BX03, C01DA02, C04AB01, C05AE03, B01AC09, and G04CA03.

### Cardiovascular Risk Grouping

Patients were classified using the atherosclerotic CVD (ASCVD) risk score and cardiovascular-related comorbidities, as used in the ACC/AHA guidelines. Individuals were considered to be at high risk if they had a 10-year ASCVD risk score 10% or greater and/or with evidence of CVD or peripheral arterial disease. Individuals with a 10-year ASCVD risk score less than 10% or who had a previous stroke were classified as low to moderate risk using the ACC/AHA guidelines. To provide a comparison in a Canadian context, a mean Framingham risk score was also reported. Both the ASCVD and Framingham risk scores were calculated using published algorithms.^33,34^ Owing to the limited reporting of ethnicity in the CPCSSN database, the ASCVD algorithms for white individuals were used. Patients with missing data required for determining the ASCVD or Framingham risk scores were omitted from the calculations of those scores.

### Statistical Analysis

Prevalence calculations were age and sex adjusted to the Canadian population using the 2016 census to reflect national population-level estimates.^35^ The proportions of individuals classified as having hypertension, receiving or eligible for treatment with an antihypertensive medication, and achieving target BP control determined using criteria and risk groupings from the ACC/AHA and Hypertension Canada guidelines were presented along with corresponding 95% confidence intervals based on the exact binomial distribution. Analyses were performed using R, version 3.3.1 (R Foundation Inc).

## Results

### Hypertension Diagnosis

A cohort of 594 492 Canadian participants with at least 1 primary care visit in the previous 2 years was assembled through the CPCSSN database. A quarter of individuals had an existing diagnosis of hypertension (144 348 [24.2%]; 45.6% male; mean [SD] age, 65.5 [14.5] years). On applying a BP cutoff of 130/80 mm Hg or greater, as proposed by the ACC/AHA guidelines, the number of people considered to have hypertension nearly doubled to 252 279 individuals, representing a prevalence of 42.4% (Table 1). The crude prevalence of hypertension increased from 13.3% to 32.0% in those aged 18 to 64 years, and of those aged 65 years and older, 16.6% more individuals were reclassified as having hypertension (from 55.2% to 71.8%). Canadian patients who were not previously considered to have hypertension but were reclassified as having elevated BP using the lower cutoff of 130/80 mm Hg or greater, when compared with those with an established diagnosis of hypertension, tended to be younger (mean [SD] age, 50.7 [16.2] vs 65.5 [14.5] years) and male (54 594 [50.6%] vs 65 777 [45.6%]) and were less likely to have an existing prescription for antihypertensive drug treatment (11.5%; 95% CI, 11.3%-11.7% vs 80.5%; 95% CI, 80.3%-80.7%); they also had fewer comorbid conditions, such as CVD (1211 [1.1%] vs 9638 [6.7%]), stroke (441 [0.4%] vs 3120 [2.2%]), congestive heart failure (116 [0.1%] vs 1650 [1.1%]), chronic kidney disease (695 [0.6%] vs 5058 [3.5%]), diabetes (10 578 [9.8%] vs 39 277 [27.2%]), and obesity (26 919 [24.9%] vs 40 377 [28.0%]), and generally had lower 10-year ASCVD risk scores (mean [SD], 7% [13%] vs 23% [23%]).

**Table 1.** Patient Characteristics of Individuals With Hypertension Before and After Applying the 2017 ACC/AHA Blood Pressure Guidelines

Patient Characteristic	No. (%)		
	Patients With Hypertension in the CPCSSN Database (n = 144 348)	Patients With Hypertension Using the ACC/AHA Guidelines (n = 252 279)	Reclassified Patients (n = 107 931)
Age, mean (SD), y	65.5 (14.5)	59.2 (16.9)	50.7 (16.2)
Age ≥65 y	85 872 (59.5)	111 625 (44.2)	25 753 (23.9)
Male	65 777 (45.6)	120 371 (47.7)	54 594 (50.6)
Urban residence	108 918 (75.5)	193 730 (76.8)	84 812 (78.6)
BMI ≥30 (obese)	40 377 (28.0)	67 296 (26.7)	26 919 (24.9)
CVD	9638 (6.7)	10 849 (4.3)	1211 (1.1)
Stroke	3120 (2.2)	3561 (1.4)	441 (0.4)
CHF	1650 (1.1)	1766 (0.7)	116 (0.1)
CKD	5058 (3.5)	5753 (2.3)	695 (0.6)
Diabetes	39 277 (27.2)	49 855 (19.8)	10 578 (9.8)
10-y Framingham risk score, mean (SD), %	21 (9)^a^	19 (10)^b^	16 (10)^c^
10-y ASCVD risk score, mean (SD), %	23 (23)^a^	17 (21)^b^	7 (13)^c^
≥3 BP measurements recorded in previous 2 y	102 664 (71.1)	147 691 (58.5)	45 027 (41.7)
No BP measurements in previous 2 y	12 851 (8.9)	12 851 (5.1)	0

Abbreviations: ACC/AHA, American College of Cardiology/American Heart Association; ASCVD, atherosclerotic cardiovascular disease; BMI, body mass index (calculated as weight in kilograms divided by height in meters squared); BP, blood pressure; CHF, congestive heart failure; CKD, chronic kidney disease; CPCSSN, Canadian Primary Care Sentinel Surveillance Network; CVD, cardiovascular disease.

^a^ Missing information for 44 826 of 99 522 patients.

^b^ Missing information for 89 199 of 163 080 patients.

^c^ Missing information for 44 373 of 63 558 patients.

Of the 107 931 individuals reclassified as having hypertension, most of the participants (82 178 [76.1%]) were younger than 65 years, and relatively few were at high cardiovascular risk (13 251 [12.3%]). The prevalence of hypertension increased 2.5-fold for younger individuals (age 18-64 years), compared with only 30% for older persons (age ≥65 years) with the institution of the ACC/AHA guidelines (Table 2). After standardizing to the Canadian population for age and sex, our prevalence estimates remained similar overall, although the adjusted prevalence when stratified by age was slightly higher than the crude.

**Table 2.** Differences in Hypertension Prevalence, Treatment, and Control Rates Before and After Applying the 2017 ACC/AHA Blood Pressure Guidelines

Characteristic	% (95% CI)		
	Patients With Established Hypertension in Canada	Patients With Hypertension Using the ACC/AHA Guidelines	Reclassified Patients
Patient population, No.	(n = 144 348)	(n = 252 279)	(n = 107 931)
Crude prevalence	24.2 (24.0-24.4)	42.4 (42.2-42.6)	
Age- and sex-adjusted prevalence	24.4 (24.2-24.6)	42.9 (42.7-43.1)	
Proportion prescribed antihypertensive medication	80.5 (80.3-80.7)	51.0 (50.8-51.2)	11.5 (11.3-11.7)
Proportion achieving BP targets	58.2 (57.9-58.4)	41.1 (41.0-41.3)	90.2 (90.1-90.4)
Proportion of patients on medication and achieving BP targets	59.5 (59.2-59.8)	43.5 (43.2-43.7)	90.5 (90.0-91.0)
Age 18-64 y	(n = 58 476)	(n = 140 654)	(n = 82 178)
Crude prevalence	13.3 (13.0-13.6)	32.0 (31.8-32.2)	
Age- and sex-adjusted prevalence	15.8 (15.5-16.1)	35.1 (34.9-35.4)	
Proportion prescribed antihypertensive medication	77.1 (76.7-77.4)	36.8 (36.6-37.1)	8.2 (8.0-8.4)
Proportion achieving BP targets	59.2 (58.8-59.6)	43.2 (43.0-43.5)	89.1 (88.9-89.3)
Proportion of patients on medication and achieving BP targets	60.9 (60.4-61.3)	45.5 (45.1-45.9)	87.2 (86.4-88.0)
Age ≥65 y	(n = 85 872)	(n = 111 625)	(n = 25 753)
Crude prevalence	55.2 (54.9-55.5)	71.8 (71.5-72.1)	
Age- and sex-adjusted prevalence	60.1 (59.8-60.4)	74.9 (74.6-75.2)	
Proportion prescribed antihypertensive medication	82.8 (82.5-83.0)	68.8 (68.5-69.0)	22.0 (21.5-22.5)
Proportion achieving BP targets	57.5 (57.2-57.8)	38.5 (38.3-38.8)	93.8 (93.5-94.1)
Proportion of patients on medication and achieving BP targets	58.6 (58.2-58.9)	42.1 (41.8-42.5)	94.5 (93.9-95.1)
High-risk group (all ages)^a^	(n = 67 770)	(n = 81 021)	(n = 13 251)
Proportion of hypertensive patients in high risk group	46.9 (46.5-47.3)	32.1 (31.8-32.4)	12.3 (11.7-12.9)
Proportion prescribed antihypertensive medication	92.9 (92.7-93.1)	85.8 (85.5-86.0)	49.1 (48.2-49.9)
Proportion achieving BP targets	60.1 (59.8-60.5)	42.0 (41.7-42.4)	92.8 (92.3-93.2)
Proportion of patients on medication and achieving BP targets	60.5 (60.1-60.9)	43.9 (43.6-44.3)	92.2 (91.5-92.9)

Abbreviations: ACC/AHA, American College of Cardiology/American Heart Association; BP, blood pressure.

^a^ High-risk group defined according to 2017 ACC/AHA guidelines as atherosclerotic cardiovascular disease 10% or greater or cardiovascular disease or peripheral artery disease.

### Pharmacological Treatment

Most patients with hypertension in Canada were prescribed an antihypertensive medication (80.5%; 95% CI, 80.3%-80.7%), compared with only half of participants defined by the ACC/AHA guidelines (51.0%; 95% CI, 50.8%-51.2%) (Table 2). Older adults (age ≥65 years) with hypertension, when compared with younger individuals (age 18-64 years), were more likely to receive prescriptions for antihypertensive drugs (82.8%; 95% CI, 82.5%-83.0% vs 77.1%; 95% CI, 76.7%-77.4%, respectively). Within the high-risk group, nearly all (92.9%; 95% CI, 92.7%-93.1%) Canadian patients with an existing diagnosis of hypertension received a prescription for an antihypertensive medication, but the proportion of treated patients decreased to 85.8% (95% CI, 85.5%-86.0%) when the ACC/AHA diagnostic and treatment thresholds were applied.

### Blood Pressure Control

Overall, the proportion of patients achieving BP targets was higher in the Canadian cohort (58.2%; 95% CI, 57.9%-58.4%) than in the ACC/AHA cohort (41.1%; 95% CI, 41.0%-41.3%) (Table 2). Among those prescribed an antihypertensive medication, BP control rates remained higher in the Canadian cohort (59.5%; 95% CI, 59.2%-59.8%) compared with the ACC/AHA group (43.5%; 95% CI, 43.2%-43.7%). Less than 10% of patients who were reclassified as having high BP with the ACC/AHA guidelines but were not previously considered to have hypertension had BP levels above the suggested ACC/AHA treatment targets, and thus potentially eligible for treatment initiation or intensification. Younger adults (age 18-64 years) were slightly more likely to achieve target BP control compared with those older than 65 years. In high-risk individuals, BP control was achieved by a larger proportion of patients in the Canadian group (60.1%; 95% CI, 59.8%-60.5%) compared with the ACC/AHA (42.0%; 95% CI, 41.7%-42.4%).

## Discussion

The BP levels used to define hypertension proposed by the ACC/AHA guidelines are associated with potentially important changes for clinical care and public health.^9,20,36^ We found that lowering the BP cutoff to 130/80 mm Hg or greater for diagnosis would nearly double the prevalence of hypertension in Canada from 24.2% to 42.4%. It was estimated that 5.2 million Canadian adults would be reclassified from nonhypertensive to hypertensive status (based on estimates from the 2016 Canadian census).^35^ Most of the individuals associated with this change would be younger and considered to have a normal BP using traditional thresholds (ie, <140/90 mm Hg). Many of the people who were reclassified as having hypertension were at low to moderate cardiovascular risk.

Our findings are consistent with previous studies. In a cross-sectional study using the US National Health and Nutrition Examination Survey from 2011 to 2014, Muntner and colleagues^20^ found that redefining hypertension using BP levels 130/80 mm Hg or greater, compared with 140/90 mm Hg or greater, would lead to a considerable increase in the prevalence of hypertension from 32% to 46% of US adults. Approximately 5% of treatment-naive individuals would be eligible for pharmacological treatment and another 29% of adults would be reclassified as having uncontrolled hypertension, thus requiring escalation of treatment.^20^ Nearly half of US patients potentially affected by the new definition of hypertension were younger than 55 years.^20^ In contrast, relatively few Canadian patients would require further treatment intensification to meet US BP targets, likely because Canadian guidelines already incorporate recommendations for lower treatment targets in selected high-risk individuals (SBP ≤120 mm Hg), as well as those with diabetes (<130/80 mm Hg) (see eTable in the Supplement for comparison between ACC/AHA and Canadian blood pressure guidelines).^10^

Compelling evidence from randomized clinical trials demonstrates that BP decrease effectively prevents cardiovascular events and death.^37^ The absolute benefits of BP lowering are most evident in individuals at higher cardiovascular risk, as well as those with higher baseline BP levels.^37,38,39^ However, as modest BP elevations are much more prevalent than severe elevations in the population, even small shifts in BP thresholds and targets tend to result in large changes in the number of people who become medicalized but may be associated with a comparatively small absolute risk reduction. Since the diagnosis of hypertension often results in a permanent label, the rationale for medicalizing these individuals needs to be appropriately justified and the benefits should outweigh foreseeable harm. The ACC/AHA guidelines place emphasis on healthy lifestyle behaviors, which are important for all and should be encouraged with every clinical encounter. However, mass medicalization may also lead to unintended costs to patients, clinicians, and the health care system. Labels of disease may generate negative perceptions of self-identity, particularly among young adults^40^; patients with hypertension tend to have more primary care visits compared with nonhypertensive individuals^27^; and, an increase in health care encounters may be challenging to accommodate, as low-risk patients potentially take away limited time and resources from higher-risk patients who need to be seen the most. Importantly, balancing the trade-off between treatment benefits and adverse drug events becomes significantly less favorable when treatment is expanded to include relatively low-risk patients with diminishing benefits in lower-risk groups.^41^ Indeed, there is little evidence to support the benefit of treatment in individuals with SBP less than 140 mm Hg without CVD or risk factors.^38,39,42^

The strongest support for lower BP targets comes from SPRINT, a randomized clinical trial of 9631 individuals at high risk for CVD (but without diabetes, prior stroke, or congestive heart failure).^7^ Intensive treatment (targeting SBP <120 mm Hg), compared with standard control (SBP <140 mm Hg), resulted in a significant decrease in major cardiovascular events by 25% (absolute event rate per year of 2.19% vs 1.65%).^7^ Although seemingly impressive, these results are not applicable to all patients with hypertension. First, participants in SPRINT were selected based on their high cardiovascular risk and older age (mean age, 68 years). The benefits of BP lowering in SPRINT have not been conclusively demonstrated in individuals with low to intermediate cardiovascular risk or those with diabetes.^43,44,45^ Second, SPRINT used an unattended automated BP measurement technique, a method that often yields SBP values 10 to 20 mm Hg lower than traditional attended office BP readings.^46^ Finally, implementation of an intensive treatment strategy resulted in more clinical encounters, monitoring, and medication use. In SPRINT, participants were followed up monthly until target BP levels were achieved and were prescribed, on average, 2.7 antihypertensive drugs (compared with 1.8 in the control group).^7^ The treatment gains reported in the trial may not necessarily be generalizable to younger or lower-risk groups, and the study protocol that was used to achieve lower BP levels may be difficult to practically implement in many clinical settings.

Our findings must be interpreted in the context of the study design. While most Canadian estimates of hypertension prevalence have come from cross-sectional surveys and administrative data,^21,47^ we used a national primary care EMR data repository to estimate prevalence rates with age and sex standardization according to the Canadian population. In contrast to surveys (which may be prone to selection, reporting, and recall bias) or administrative data (which often lack clinical and contextual information), a cardinal strength of the CPCSSN database is its rich and longitudinal real-world clinical information, as well as its nationally validated case-finding algorithms developed for various chronic diseases.^26^ Additionally, since most Canadian individuals visit their general practitioner each year,^48^ the CPCSSN database can be a cost-efficient and expeditious source for conducting hypertension surveillance in the Canadian population.

### Limitations

Our study had limitations. First, data were collected for clinical (not research) purposes and some relevant variables may not have been consistently documented. Still, we found that most patients (>90%) with a clinic visit in the previous 2 years had at least 1 BP measurement recorded. Second, BP measurements were not standardized according to technique (eg, attended vs unattended) or device (eg, automated vs manual), and these variations can lead to differences in recorded BP levels (eg, 10-20/5-15 mm Hg).^46,49,50,51,52^ Third, treatment with antihypertensive medication was based on prescribing information. We could not confirm medication dispensation or adherence. Fourth, the definitions used for defining some comorbidities have not been validated for a primary care EMR database (apart from hypertension and diabetes) and may be subject to misclassification. Fifth, cardiovascular risk estimates could be calculated only for patients with complete data for all pertinent variables. For example, missing data for smoking status was a main limiting factor when calculating cardiovascular risk scores and, subsequently, risk scores were not calculated in 31% of patients in the Canadian cohort and 35% of patients in the ACC/AHA cohort. Accordingly, this may have led to an overestimation of cardiovascular risk, as less healthy patients may be more likely to have certain data recorded (eg, smoking status and laboratory measures).

## Conclusions

Redefining the definition of hypertension according to BP levels 130/80 mm Hg or greater and revising the BP goal to target less than 130/80 mm Hg may result in a considerable change in the epidemiology of hypertension, with an estimated 5.6 million people being reclassified from nonhypertensive to hypertensive in Canada, most of whom are at low cardiovascular risk. A focus on lower BP thresholds and targets may also potentially increase the absolute number of people eligible for treatment but is likely associated with a relatively small change in risk for most individuals. Implementation of the proposed ACC/AHA BP guidelines in Canada may have a major effect on patients, resource use, policy, and health care delivery.

## Notes

Supplement. **eTable.** Blood Pressure Thresholds for Initiation of Antihypertensive and Treatment Targets in Adults According to the 2018 Hypertension Canada Guidelines and the 2017 ACC/AHA **eReferences**
