# Supplementary material for: Prevalence of Hypertension, Treatment, and Blood Pressure Targets in Canada Associated With the 2017 American College of Cardiology and American Heart Association Blood Pressure Guidelines
Source: JAMA Netw Open. 2019 Mar 8;2(3):e190406. doi: 10.1001/jamanetworkopen.2019.0406 (PMC6484648; doi:10.1001/jamanetworkopen.2019.0406)
Supplement: Supplement. — eTable. Blood Pressure Thresholds for Initiation of Antihypertensive and Treatment Targets in Adults According to the 2018 Hypertension Canada Guidelines and the 2017 ACC/AHA eReferences [file jamanetwopen-2-e190406-s001.pdf]

Supplementary Online Content

Garies S, Hao S, McBrien K, et al; Hypertension Canada's Research and Evaluation Committee. Prevalence of Hypertension, treatment, and blood pressure targets in Canada associated with the 2017 American College of Cardiology and American Heart Association blood pressure guidelines. *JAMA Netw Open*. 2019;2(3):e190406. doi:10.1001/jamanetworkopen.2019.0406

eTable. Blood Pressure Thresholds for Initiation of Antihypertensive and Treatment Targets in Adults According to the 2018 Hypertension Canada Guidelines and the 2017 ACC/AHA
eReferences

This supplementary material has been provided by the authors to give readers additional information about their work.

© 2019 Garies S et al. *JAMA Network Open*.

eTable. Blood Pressure Thresholds for Initiation of Antihypertensive and Treatment Targets in Adults According to the 2018 Hypertension Canada Guidelines and the 2017 ACC/AHA

**Patient Population**	**SBP/DBP threshold (mmHg) for initiation of antihypertensive therapy**		**SBP/DBP target (mmHg) for treatment**	
	**ACC/AHA**	**HC**	**ACC/AHA**	**HC**
Low-risk (e.g., no target organ damage or cardiovascular risk factors)	SBP ≥140<br>DBP ≥90	SBP ≥160<br>DBP ≥100	SBP <130<br>DBP <80	SBP < 140<br>DBP < 90
High-risk of cardiovascular disease	SBP ≥130<br>DBP ≥80	SBP ≥130[a]	SBP <130<br>DBP <80	SBP <120[a]
Diabetes mellitus	SBP ≥130<br>DBP ≥80	SBP ≥130<br>DBP ≥80	SBP <130<br>DBP <80	SBP <130<br>DBP <80

Abbreviations: ACC/AHA=American College of Cardiology / American Heart Association; DBP=diastolic blood pressure; HC=Hypertension Canada; SBP=systolic blood pressure.

[a]Using an automated office blood pressure measurement.

© 2019 Garies S et al. *JAMA Network Open*.

eReferences

1. Whelton PK, Carey RM, Aronow WS, et al. 2017 ACC/AHA/AAPA/ABC/ACPM/AGS/APhA/ASH/ASPC/NMA/PCNA Guideline for the Prevention, Detection, Evaluation, and Management of High Blood Pressure in Adults: A Report of the American College of Cardiology/American Heart Association Task Force on Clinical Practice Guidelines. J Am Coll Cardiol. 2018 May 15;71(19):e127-e248.
2. Nerenberg KA, Zarnke KB, Leung AA, et al. Hypertension Canada's 2018 Guidelines for Diagnosis, Risk Assessment, Prevention, and Treatment of Hypertension in Adults and Children. Can J Cardiol. 2018 May;34(5):506-525.

© 2019 Garies S et al. *JAMA Network Open*.
